# Non-mosaic trisomy 22 and congenital heart surgery using the shared decision making model: a case report

**DOI:** 10.1186/s12887-023-03949-8

**Published:** 2023-03-18

**Authors:** Vivien Phung, Kathryn E. Singh, Saar Danon, Christopher A. Tan, Sarah Dabagh

**Affiliations:** 1 Department of Pediatrics, University of California, Irvine, USA; 2 Department of Medical Genetics, Miller Women and Children’s Hospital, Long Beach, CA USA; 3 Department of Pediatric Cardiology, Miller Women and Children’s Hospital, Long Beach, CA USA; 4 Department of Pediatrics, University of California, Los Angeles, USA; 5 Department of Palliative Care, Miller Women and Children’s Hospital, Long Beach, CA USA; 6 Department of Medicine, University of California, Irvine, USA

**Keywords:** Trisomy 22, Shared Decision making, Congenital Heart defects, Case report

## Abstract

**Background:**

Liveborn infants with non-mosaic trisomy 22 are rarely described in the medical literature. Reported lifespan of these patients ranges from minutes to 3 years, with the absence of cardiac anomalies associated with longer-term survival. The landscape for offering cardiac surgery to patients with rare autosomal trisomies is currently evolving, as has been demonstrated recently in trisomies 13 and 18. However, limited available data on patients with rare autosomal trisomies provides a significant challenge in perinatal counseling, especially when there are options for surgical intervention.

**Case presentation:**

In this case report, we describe an infant born at term with prenatally diagnosed apparently non-mosaic trisomy 22 and multiple cardiac anomalies, including a double outlet right ventricle, hypoplastic aortic valve and severe aortic arch hypoplasia, who underwent cardiac surgery. The decisions made by her family lending to her progress and survival to this day were made with a focus on the shared decision making model and support in the prenatal and perinatal period. We also review the published data on survival and quality of life after cardiac surgery in infants with rare trisomies.

**Conclusions:**

This patient is the only known case of apparently non-mosaic trisomy 22 in the literature who has undergone cardiac surgery with significant survival benefit. This case highlights the impact of using a shared decision making model when there is prognostic uncertainty.

## Article summary

Case report detailing shared decision making for cardiac surgery in trisomy 22, including the first reported infant to survive cardiac surgery to discharge.

## Background

Trisomy 22, while one of the most common trisomies identified in spontaneous abortions, is extremely rare in liveborns and scantly described in the literature as most fetuses with this condition do not survive to birth [1]. In those who do survive, it is even more rare to have a complete, non-mosaic form. Clinical features of trisomy 22 include, but are not limited to, intrauterine growth restriction, microcephaly, broad nasal bridge, epicanthal folds, micrognathia, and long philtrum, abnormally shaped/positioned ears, webbed neck or redundant skin, cleft lip/palate, congenital heart defects, and genitourinary abnormalities [2]. The phenotype varies more in individuals with mosaic trisomy 22. In previous reports, the lifespan of individuals with non-mosaic trisomy 22 has ranged from minutes to years, with median survival of 4 days and rare survival beyond the first 2 weeks of life [1]*.* There are occasional cases in the literature that describe survival up to nearly 3 years of age [3], however these cases are not typically associated with cardiac anomalies. It is likely that there are other individuals with potentially longer lifespan but are not represented in the literature.

The current landscape of congenital heart repair in patients with rare trisomies is evolving, with an increasing number of centers offering this option [4]. While this aspect of care for babies with trisomy 18 and 13 is challenging even with a comparative abundance of literature on survival, the lack of information on trisomy 22 provides even more of a prognostic challenge in prenatal and perinatal counseling. Shared decision making (SDM) has been proposed as a model to guide families in making informed, goals-focused decisions when faced with difficult decisions [5]. This model includes the healthcare team comprehensively reviewing medical information with patients and their families, supporting them in considering options, and jointly arriving at a clinical decision [5]. We describe a term infant with prenatally diagnosed apparently non-mosaic trisomy 22 who underwent congenital heart surgery, with a focus on the SDM model and support in the perinatal period.

## Case presentation

We describe the case of a term female infant with prenatal diagnosis of trisomy 22 diagnosed at 26 weeks gestation by amniocentesis. Prenatal ultrasound showed cardiac anomalies (VSD, possible overriding aorta, small left outflow tract), agenesis of the corpus callosum, unilateral microphthalmia, and bilateral cleft lip and palate. Amniocentesis was offered, and karyotype demonstrated three separate copies of chromosome 22 along with an unrelated paternally inherited balanced 5;6 translocation with no evidence of mosaicism (15 metaphases counted). Chromosomal microarray showed one of the copies of 22 has a 2.05 Mb (approximately 4% of the total material on chromosome 22) interstitial deletion at 22q13.2 ([hg19] 41,422,990–43,473,370). Mother had targeted qPCR for the deleted region and had two copies, but father was not tested. Based on the small size of this deletion, the fetus was considered to have complete trisomy 22. Through the hospital’s perinatal program, the family received genetic counseling, palliative care, cardiology and cardiothoracic surgery referrals. Using SDM with the palliative care team, the family expressed their desire to meet their child alive. Their plan included vaginal delivery, and if indicated, cesarean section and routine neonatal resuscitation including intubation. It was discussed with the family that their baby’s future was uncertain, but that if she survived after delivery, she would require multiple cardiac surgeries for longer term survival.

**Fig. 1:**
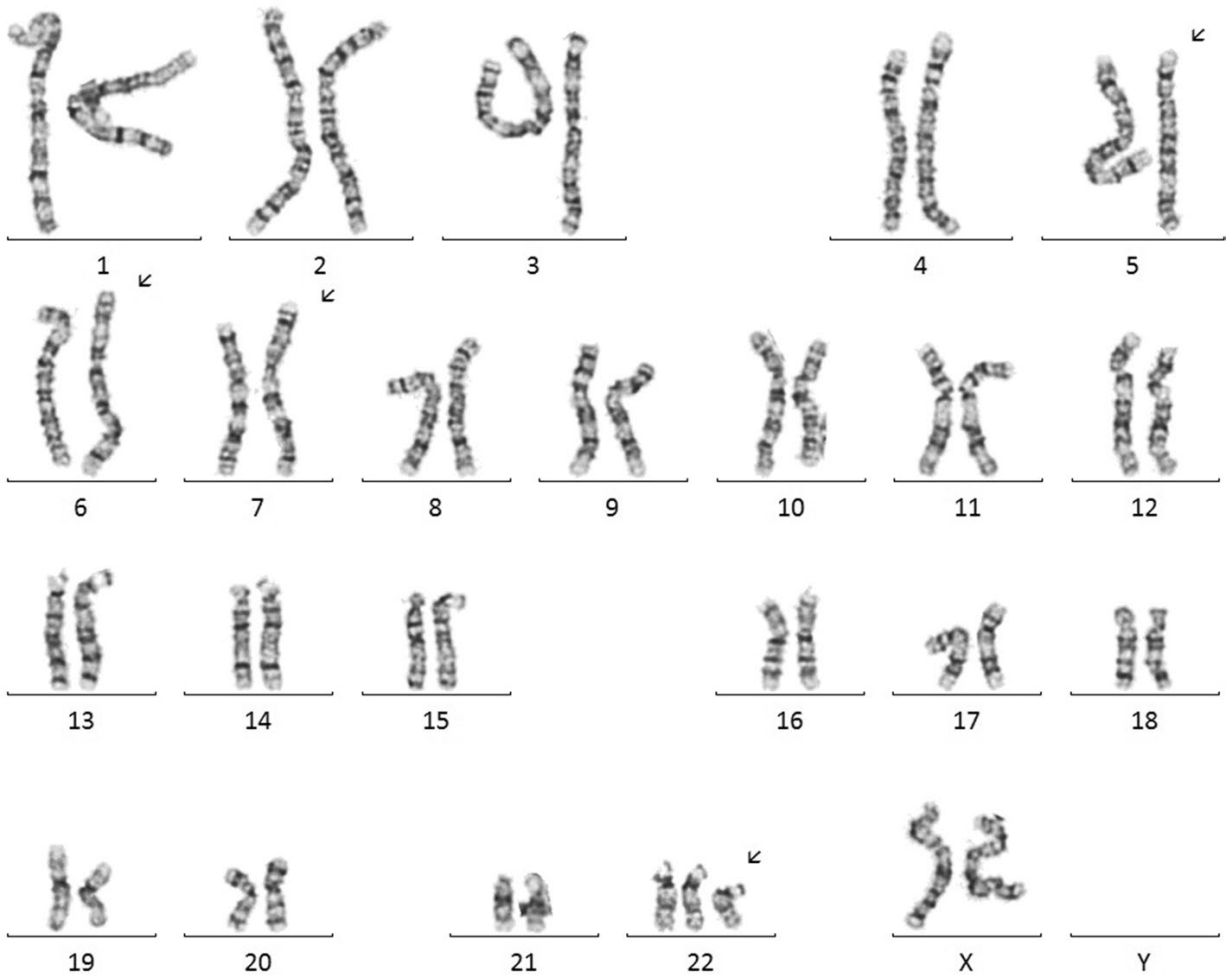
Karyotype demonstrating Trisomy 22. This karyotype results demonstrating complex chromosome abnormality, with abnormalities of four different chromosomes. Most notable is trisomy 22, but the infant also has an apparently balanced translocation involving the long arms of chromosomes 5 and 6, as well as a subtle inversion on the short arm of chromosome 7. The 2 Mb deletion on one of the copies of chromosome 22 was not visible by karyotype but was identified on chromosomal microrarray (not shown). Arrows indicate abnormal chromosomes

The infant was born at 39 weeks and 3 days gestation with a birthweight of 2010 g and Apgar score of 6 and 9. After resuscitation, per the family’s birth plan, the infant received skin-to-skin with mother for 10 min prior to transfer to the NICU. The infant’s exam was notable for a large fontanelle, midface hypoplasia, bilateral cleft lip and palate, low-set ears, preauricular ear pits, wide-spaced eyes and likely microphthalmia, wide-spaced nipples, bulbous fingertips, and slight spacing between 1st and 2nd toes. Tone was normal with intact reflexes including a vigorous suck reflex. The infant was started on prostaglandin at 0.025mcg/kg/min and echocardiogram demonstrated double outlet right ventricle (DORV) with a doubly committed VSD, ASD, hypoplastic aortic valve and arch hypoplasia. Abdominal ultrasound was unremarkable. Brain MRI demonstrated dysgenesis of the corpus callosum. Postnatal blood karyotype confirmed trisomy 22 without evidence of mosaicism (50 cells counted), confirmed the 5;6 translocation, and also identified a subtle paracentric inversion in the short arm of chromosome 7 (Fig. 1). Additional tissue types were not analyzed so mosaicism cannot be ruled out in other tissues, but given trisomy 22 in all 50 cells analyzed in blood, the disorder was clinically considered to be non-mosaic.

A multidisciplinary family meeting was arranged in order to revisit the infant’s plan of care with the information the team had obtained thus far. Prior to this meeting, the family met with the perinatal coordinator and palliative care team. The family was given a space to continue to process the grief of a normal pregnancy, the joy of meeting their child alive, and their hopes and worries moving forward. Discussion was tailored to further clarify family values, solidify questions the family was hoping to find answers for, reaffirm which family members were key for decision making, and identify key psychosocial providers who would be available for support.

**Fig. 2:**
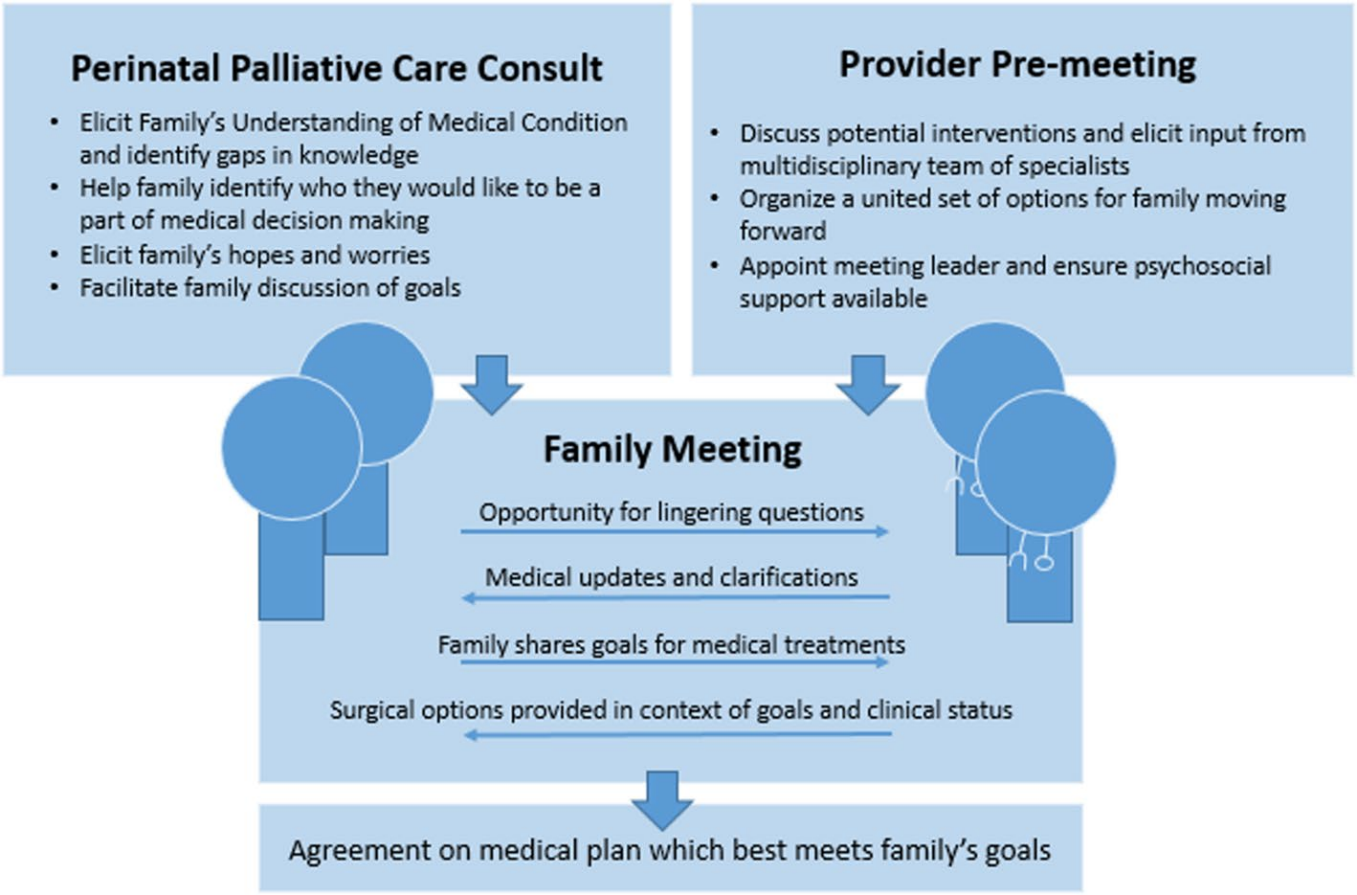
Shared Decision Making Model. Shared Decision Making is a model through which clinicians collaborate with families to reach evidence-informed and value-congruent decisions regarding medical interventions. When multiple of the available choices are ethically equivalent and there exists uncertainty regarding outcomes, an emphasis is placed on value-congruent care and family participation in decision making. Preparatory meetings, both within the family unit and within the medical team, can help streamline decision making in this model as demonstrated above

In the larger multi-team family meeting, using a SDM model (Fig. 2), the separate paths forward were described for this family, including a pathway with a focus on comfort and a pathway with a focus on time. It was made clear that the infant may not survive the perioperative period, but there was potential for prolongation of life expectancy and reasonable expectation for quality of life. The discussion included the fact that the infant was not a candidate for medical extracorporeal membrane oxygenation (ECMO) but would qualify for surgical ECMO in the case that she would require it during or immediately after the surgery. The approach of performing a staged repair was recommended by the cardiothoracic team based on the infant’s intracardiac anatomy. The family was able to voice back this understanding and shared with the team a goal for maximum time, understanding those risks, and decided on pursuing cardiac surgery.

**Fig. 3:**
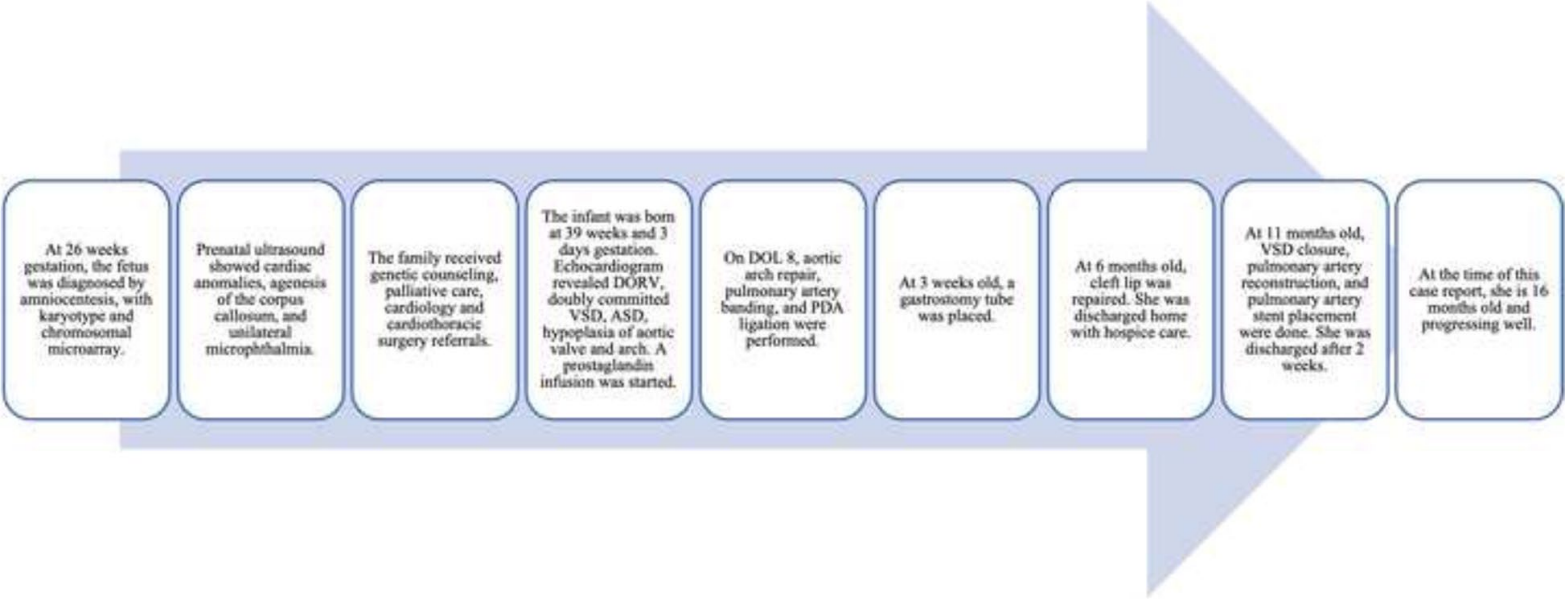
Timeline of care

Aortic arch repair, pulmonary artery banding, and PDA ligation was performed on DOL 8, followed by a separate procedure for gastrostomy tube placement due to the patient’s cleft palate causing potential for aspiration with oral feedings. Following an approximately 3 week stay in the ICU she was able to be discharged home with the support of concurrent care hospice. She subsequently had complete surgical repair at 11 months of age, consisting of VSD closure and pulmonary artery reconstruction, followed by cardiac catheterization with pulmonary artery stent placement (Fig. 3). She had persistent fevers that required prolonged antibiotics, but ultimately discharged home. At the time of this case report, she is 16 months old and progressing well.

## Discussion and conclusions

While many families will choose a focus on comfort, knowing that long-term survival is unlikely, some may choose to pursue life-sustaining surgeries. The patient described in this report has already exceeded the life expectancy suggested by previous case reports, though notably she is only the second patient with trisomy 22 in the literature to undergo cardiac repair, and the first to demonstrate significant survival benefit. This outlines a particular challenge in prenatal and perinatal counseling: How do we guide families in a SDM model based on their personal values, when outcomes are uncertain and there is a lack of available supporting data?

In 1982, a landmark case involving parents of an infant with trisomy 21 who chose to defer a surgery in favor of comfort care was one of the events behind ethics committees endorsing SDM [5]. When uncertainty exists in prognostication, the practice of patient-centered care and SDM has been the supported approach for parents and clinicians to use [5]. In this particular case, as the family was able to obtain the diagnosis prenatally in the second trimester, they had time to process, gather knowledge and discuss their options with a wide range of medical specialties.

There has been a cultural shift in the landscape of congenital cardiac surgery in patients with rare trisomies, with more centers beginning to offer congenital heart surgeries, both palliative and reparative [6], in addition to the offer of comfort focused care. While research suggests the vast majority of cardiologists support parents who would choose not to pursue surgical intervention for patients with trisomy 13 and 18 [7], more centers are beginning to offer repair, with some notable examples such as Children’s Hospital and Medical Center of Omaha.

The focus in the literature has been on trisomies 13 and 18, with little information available to help guide clinicians and families in prognosticating for rarer major chromosome abnormalities. In Table 1, we review the published cases of patients with non-mosaic trisomy 22 and congenital heart disease. Interestingly, many of the diagnoses, including our patient, involve conotruncal abnormalities, which are also seen in individuals with a small deletion of the same chromosome (22q11.2 deletion syndrome).

**Table 1:** Trisomy 22 cases without surgical repair

Reference	Sex	Congenital Heart Defect	Survival
Voiculescu et al. [1987] [8]	F	TOF with pulmonary atresia, LSVC, hypoplastic IVC^a^	10 min
McPherson and Stetka [1990] [9]	M	AVSD	33 days
Phillipson et al. [1990] [10]	M	ASD, VSD, BAV	20 h
Phillipson et al. [1990] [9]	M	Tricuspid atresia, VSD, severe aortic stenosis	4 h
Stratton et al. [1993] [11]	F	TOF^a^	4 months
Fahmi et al. [1994] [12]	F	ASD, VSD, ARSCA	4 months
Nicholl et al. [1994] [13]	M	ASD, possible abnormal pulmonary valve	3 months
Bacino et al. [1995] [14]	F	HRH, VSD	2 months
Ladonne et al. [1996] [15]	F	TOF with pulmonary atresia^a^	Minutes
Manasse et al. [2000] [16]	F	ASD, VSD	18 months
Miura et al. [2000] [17]	M	ASD, VSD, PAPVR, COA, Interrupted IVC, ARSCA	10 h
Miura et al. [2000] [17]	F	ASD, HRH	3.5 months
Stressig et al. [2005] [18]	F	ASD, VSD, TV abnormality	1 day
Mihci et al. [2007] [19]	F	ASD	1 day
Barseghyan et a. [2009] [20]	M	DORV, VSD, IAA^a^	2 days
Fruhman et al. [2011] [21]	F	ASD, VSD, dysplastic AV, hypoplastic ascending aorta	3 days
Heinrich et al. [2013] [2]	M	Pulmonary stenosis, aortic stenosis	29 days
Naicker and Aldous [2013] [22]	F	ASD, VSD	2 months
Naicker and Aldous [2013] [22]	F	VSD with overriding aorta (TOF variant)^a^	11 days
Kehinde et al. [2014] [23]	F	TOF with pulmonary atresia, interrupted IVC^a^	35 days

^a^Conotruncal abnormalities

Survival data on patients with trisomy 13 and 18 may allow for some guidance for clinicians and parents of infants with the rarer trisomy 22. Notably, life expectancy in the group that did not undergo cardiac surgery ranges from a few minutes to 18 months; with a median life expectancy of 20 days. Only one other patient underwent cardiac repair (Table 2), and unfortunately died on postoperative day 6 from complications of infection and septic shock [1]. Retrospective reviews of patients with trisomy 13 and 18 who underwent congenital heart surgery demonstrated longer median survival rates than previously expected, 14.8 years for trisomy 13 and 16.2 years in trisomy 18 respectively, suggesting that survival data is a moving target as more interventions become available to these patients [24–26].

**Table 2:** Trisomy 22 cases with surgical repair

Reference	Sex	Congenital Heart Defect	Cardiac surgery	Survival
Tinkle et al. [2003] [1]	F	DORV with subaortic VSD, pulmonic/subpulmonic stenosis, ASD, bilateral SVCs^a^	BT shunt	11 weeks
*Our case*	F	DORV with a doubly committed VSD, ASD, hypoplastic aortic valve, severe aortic arch hypoplasia, large PDA^a^	Aortic arch repair, PA band and PDA ligation on DOL 8. VSD closure, ASD closure, bilateral PA plasty, with PA band removal at 11 months	Presently alive (14 months old)

^a^Conotruncal abnormalities

In addition to survival, studies show that parent-reported quality of life following cardiac repair is highly independent of the patients’ functional status [27]. These considerations of future quality of life are helpful to discuss with families who choose life-prolonging but invasive surgeries and medical technologies. Recognizing that family values and goals can often differ from provider values and goals, family perception of quality of life is a key factor in this decision making and very individualized family to family. In the case of this infant with Trisomy 22, through the gift of time of a prenatal diagnosis, multiple preparatory meetings allowed the family time to process the information, recognize and identify how they make medical decisions as a family, highlight their goals as a family, and recognize the information they felt they needed to make those decisions – including gathering and formulating questions the family could ask to get that information. This additional time, and insight from the perinatal and palliative care teams, allowed the medical team to prepare the information they would share and gather the specialists necessary to be able to answer those questions and discuss what goals might be attainable, and through what means.

Facilitating medical understanding in such a rare disorder, and paving potential paths without the support of similar case reports, was aided by a SDM model which allowed the family to align with their medical team and make an informed medical decision about the pathway forward they felt best fit with their goals and aligned with their value-based definition of quality of life.

The patient in this case report is to our knowledge the only case of apparently non-mosaic trisomy 22 in the literature who has undergone cardiac surgery and demonstrated significant survival benefit. Interestingly, she is also the second reported case of an individual with trisomy 22 and co-occurrence of an unrelated chromosome translocation [23]. While her course is not necessarily predictive of outcome for other infants with trisomy 22, families of other infants will be faced with similar decisions. This case highlights the importance of using a shared decision making model in the setting of prognostic uncertainty in the ever-evolving landscape of congenital heart repair.

## Data Availability

Data sharing not applicable to this article as no datasets were generated or analyzed during the current study.

## Abbreviations

VSD: Ventricular Septal Defect
ASD: Atrial Septal Defect
DOL: Day of Life
SDM: Shared Decision Making
NICU: Neonatal Intensive Care Unit
qPCR: Quantitative Polymerase Chain Reaction
Mb: Megabase
